# Application of Filters to Improve Flight Stability of Rotary Unmanned Aerial Objects

**DOI:** 10.3390/s22041677

**Published:** 2022-02-21

**Authors:** Maciej Salwa, Izabela Krzysztofik

**Affiliations:** Faculty of Mechatronics and Mechanical Engineering, Kielce University of Technology, Aleja Tysiąclecia Państwa Polskiego 7, 25-314 Kielce, Poland; maciejsalwa@tu.kielce.pl

**Keywords:** extended Kalman filter, complementary filter, quadrotor, PX4, MATLAB, ROS

## Abstract

The most common filters used to determine the angular position of quadrotors are the Kalman filter and the complementary filter. The problem of angular position estimation consist is a result of the absence of direct data. The most common sensors on board UAVs are micro electro mechanical system (MEMS) type sensors. The data acquired from the sensors are processed using digital filters. In the literature, the results of research conducted on the effectiveness of Kalman and complementary filters are known. A significant problem in evaluating the performance of the studied filters was the lack of an arbitrarily determined UAV position. The authors of this paper undertook the task of determining the best filter for a real object. The main objective of this research was to improve the stability of the physical quadrotor. For this purpose, we developed a research method using a laboratory station for testing quadrotor drones. Moreover, using the MATLAB environment, they determined the optimal parameters for the real filter applied using the PX4 software, which is new and has not been considered before in the available scientific literature. It should be mentioned that the authors of this work focused on the analysis of filters most commonly used for flight stabilization, without modifying the structure of these filters. By not modifying the filter structure, it is possible to optimize the existing flight controllers. The main contribution of this study lies in finding the most optimal filter, among those available in flight controllers, for angular position estimation. The special emphasis of our work was to develop a procedure for selecting the filter coefficients for a real object. The algorithm was designed so that other researchers could use it, provided they collected arbitrary data for their objects. Selected results of the research are presented in graphical form. The proposed procedure for improving the embedded filter can be used by other researchers on their subjects.

## 1. Introduction

The Kalman filter and the complementary filter are the most popular filters for determining the angular position of unmanned aerial vehicles (UAVs). The problem of angle estimation is the absence of direct data. The most common sensors on board UAVs are micro electro mechanical system (MEMS) sensors. Basic sensors are used, such as accelerometer, gyroscope, magnetometer sensors. The data acquired from the sensors are processed by using digital filters. The Kalman filter has been studied in [1,2,3,4,5,6,7]. Publications are available in which the authors have undertaken modifications to the structure of the Kalman filter. In 2015, Xiong, J.J., and Zheng, E.H. [8] developed an optimal Kalman filter (OKF) for quadrotor position estimation. In 2019, Alawsi, A.A.A., Jasim, B.H., and Raafat, S.M. [9] developed the unscented Kalman filter (UKF). In both these papers, the authors worked exclusively on simulation objects. Information about the complementary filter and its use in UAV navigation can be found in references [10,11,12,13]. The problem so far in evaluating the filter’s performance has been the lack of an arbitrary UAV position. One of the first attempts to compare the two filters was made by Walter Higgins [14]. In his 1975 paper, he compared only the theoretical operation of the filters. The authors of reference [15] used a nine-degrees of freedom (DOF) sensor and analyzed the resulting angular position from the collected readings without reference to the actual value. A reference to the arbitrary value can be found in paper [16], in which the authors attempted to represent one arm of the drone by simulating it with two motors on a ramp. They read the true value using a potentiometer. The authors of this paper did not clearly answer which of the compared filters is better. They only conclude that for quadrotors for which the flight time is no more than 15 min, initial calibration bias is not a problem for stabilization purposes. Furthermore, the simplicity of the algorithm makes the complementary filter the best choice for embedded applications where there is not much computational power.

This conclusion inspired the authors of this paper to revisit the issue, albeit using a real drone. This is an innovative approach to the UAV flight stabilization problem and has not been explored in the scientific literature.

Similar work was carried out by Stanislaw Chudzik in 2021 [17]. He studied the behavior of the filters for angle estimation for a self-balancing robot. In his research he used the STM32 Discovery F303CTV6 development kit containing MEMS sensors. He used a rotary encoder to read the actual tilt value. He too did not provide an answer as to which filter is better for tilt angle estimation. In 2016, as an extension of their research on filters used for UAVs, Gabriel Schmitz, Tiago Alves, Renato Henriques, Edison Freitas, and Ebrahim El’Youssef [18] worked on a filter for the Pixhawk controller. However, this had not been undertaken by the time the following article was written.

The authors of this paper undertook the task of determining the best filter for a real object. For this purpose, they used an innovative method for comparing the performance of filters using a drone test-bench. As a goal, they set out to answer unequivocally which filter performs better on a real object using a physical radio-controlled quadrotor. They also decided to take up a theme left uninvestigated by the aforementioned researchers; they investigated a real Pixhawk controller to determine how the default filter implemented in the PX4 software works. During their research, they developed a way to improve the performance of an extended Kalman filter, which is embedded in the PX4 software. Details concerning its differences from a standalone Kalman filter were also described. The authors determined the optimal parameters for the filter using the MATLAB environment.

This paper is structured as follows. Section 2 presents an innovative test-bench for unmanned aerial vehicle, the method of data collection including the whole algorithm in a graphical form, and the drone on which the tests were carried out. Section 3 describes the filters including mathematical relationships. Section 4 presents how the archived data were processed. Section 5 presents the results of the conducted research. Section 6 contains a discussion of the obtained results and directions for further research. Section 7 is conclusion and describes concepts for improving the performance of physical flying objects using the methods described in the paper..

## 2. Measurement Method

A real drone was used to conduct experimental research. PX4 software was uploaded to the flight controller. It enabled the drone to connect to the computer, which is the central unit of the ROS (robot operating system). The Mavlink protocol data and parameters of the drone were transmitted using a 433 MHz radio module. The Mavros program supervised the exchange of information in real time. The raw data from the sensors were archived to a csv file along with the timestamp by ROS script (written in Python). The drone was controlled via radio by the operator during the research. In the next step, the archived data were processed and filtered. This made it possible to compare the two filters under the same conditions. The raw data were recorded for all degrees of freedom in which the sensors implemented in the flight controller operate. In parallel, through the same script, data were collected concerning the physical position of the frame on which the drone was mounted.

**Figure 1:**
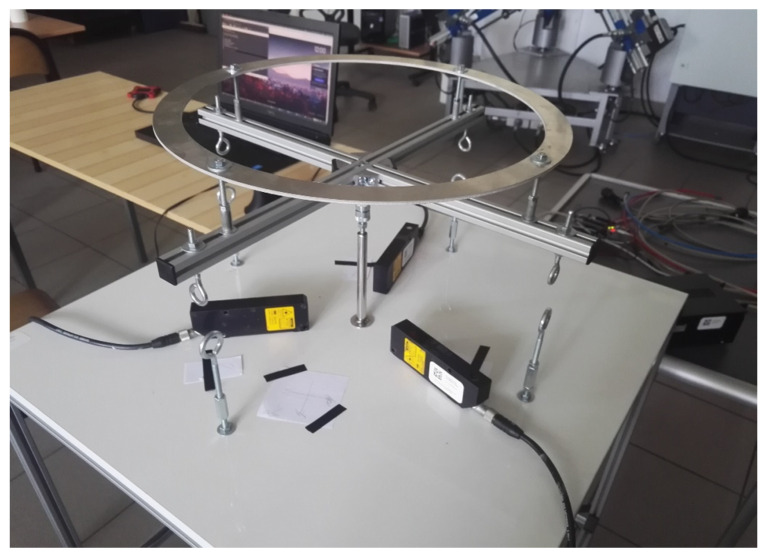
Test-bench for drones.

The information about the angular position of the frame was measured by 3 Baumer XX laser distance sensors. The sensors were spaced every 120 degrees (Figure 1). Each sensor collected data concerning the distance of the ring on which the laser beam fell. The output signal of the sensors is a voltage. The analog signals were processed using ADCs of the STM32 Nucleo-F429ZI development kit. The voltage values were divided by resistive voltage dividers to go from a 0–10 V sensor output range to a 0–3.3 V one, corresponding to the capabilities of the development board. The sensors were powered by an external 12 V power supply. Each voltage divider was made of three 470 Ohm precision resistors. The analog signals were read using a device memory access (DMA) mechanism. The microcontroller was clocked at a maximum frequency of 180 MHz. The analog readings were converted into distance values expressed in millimeters in the main program loop. The ring height was chosen to be within the sensor range of 100–600 mm. The width of the ring was chosen to be able to perform tilt and roll of the drone up to 10 degrees. This is a value consistent with the angular displacement that occurs in drones during flight, and the tests were conducted within this range. 

To convert the values from the three sensors to pitch and roll, the trigonometric relationships and the arcus tangent function were used. The pitch is calculated using the following formula:

(1) $$\varphi =arctan\left(\frac{l_{1}-\frac{\left(l_{2}+l_{3}\right)}{2}}{0.75d}\right)$$

where:

φ—pitch angle, rotation about the *x* axis,

$l_{1},l_{2},l_{3}$—the height determined by the particular sensors,

d—the diameter of the circle.

**Figure 2:**
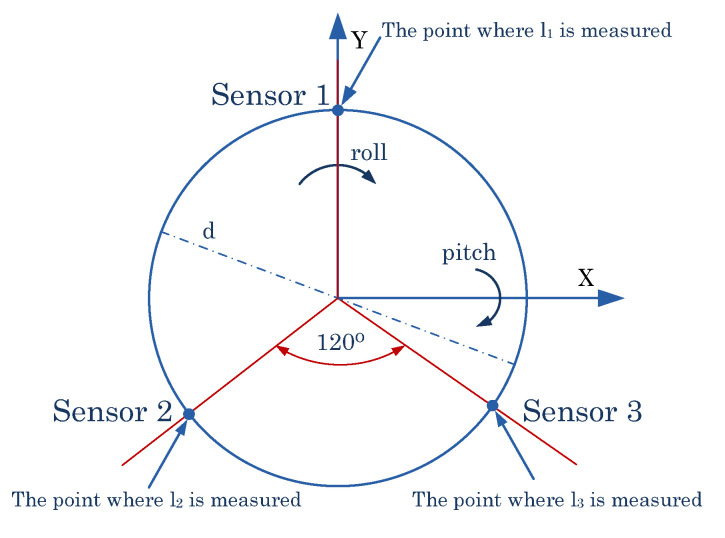
The position of the test-bench sensors in relation to the axis of the drone system.

The difference in height between the reading of sensor no. 1 and the average of readings of sensors no. 2 and 3 is divided by the height of an equilateral triangle determined by the sensors. It is a triangle inscribed in a circle (Figure 2). The pitch angle is determined by relation (1).

The roll is determined from the following formula:

(2) $$\theta =arctan\left(\frac{l_{2}-l_{3}}{\frac{\sqrt{3}}{2}\cdot d}\right)$$

where:

θ—roll angle, rotation about the *y* axis.

The difference in height between sensor no. 2 and sensor no. 3 is divided by the side of the equilateral triangle defined by the sensors. The roll angle is determined by relation (2).

The described calculations were performed in the main loop of the program running in the STM32F429 microcontroller. The positions calculated from the arcus tangent function were compensated with a logical condition so that when the drone is in an angular position deviated from the zero value in a direction not consistent with the direction of the increment, a negative value is returned. The obtained values were sent to the ROS system node via ST-link. This is a built-in programmer along with a serial communication port. Libraries prepared by the Open Agriculture Foundation were used to send the data.

An ST-link programmer was connected via USB cable to the computer on which the roscore unit was running. The rosserial library was used to receive data from the nucleo development board. Data were data published to the topic from which the archiving script subscribed. The same script in parallel subscribed to the data published by the flight controller. The writing to the csv file was carried out line by line. Raw accelerometer and gyroscope data, angular position estimated by the default Kalman filter, arbitrary values from the test-bench collected by the development kit, and timestamp were written. Data recording occurred in real time. The flight controller published the data at 50 Hz. As soon as new data became available, the script archived it along with the corresponding data from the arbitrary test-bench.

**Figure 3:**
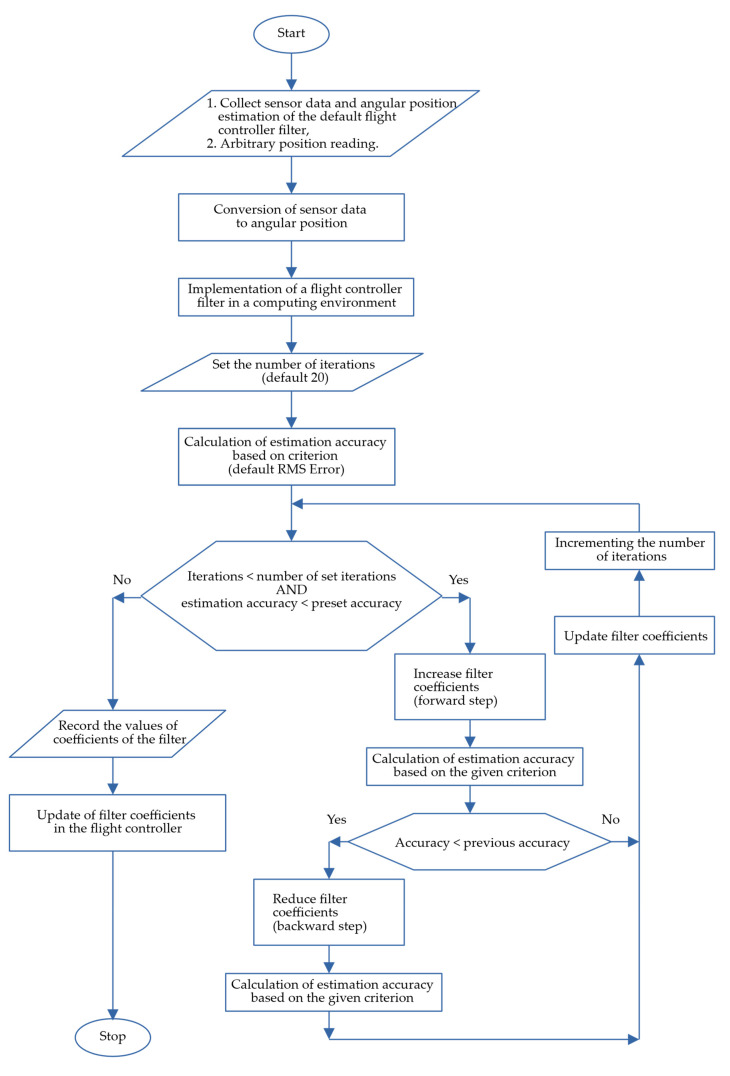
Block diagram of the filter optimization algorithm for a real flight controller.

The full algorithm of the conducted research is included below (Figure 3). 

The above diagram shows the filter coefficient optimization algorithm. The essence of this process is to collect the raw sensor data along with the arbitrary position. Then, optimization (which involves multiple iterations in the computational environment) is performed on the same data. Its effect is to obtain tuned filter coefficients, consistent in type with the filter built into the flight controller. In most flight controllers, the manufacturer’s software allows the user to enter their own filter coefficients.

### 2.1. Parameters of Drone

**Figure 4:**
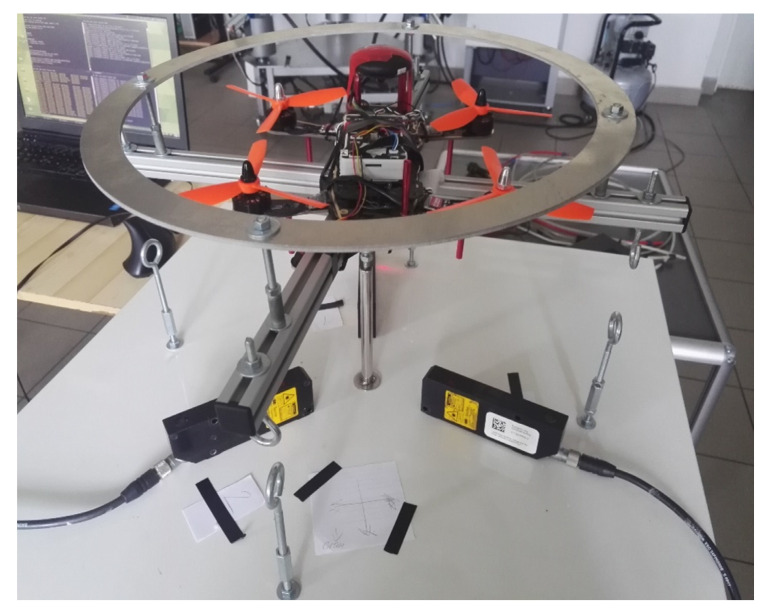
Drone mounted on the test-bench.

The selected type of drone is a quadrotor. The flight controller used is Pixhawk 2.4.8 to which PX4 software v1.12.0 was uploaded. Communication with the operator is carried out via FrSky RC radio. The drone is powered by four DC brushless motors. The K_V_ constant of the motors, which is the motor speed constant measured in revolutions per minute (RPM) per volt, or radians per volt, is equal to 2300. The sensors are located on the board the flight controller. The drone was rigidly mounted to the aluminum profiles of the test stand (Figure 4). Roll and pitch are realized by the joint on which the profiles are supported. Rotational movement of the yaw does not take place. 

The position of the test-bench was leveled in the X and Y axes before the tests started. Then, through the QGroundControl software, the zero level was set for the default Kalman filter implemented in the PX4 software. Through the mavlink protocols, the flight controller communicated with the ROS version of Melodic Morenia installed inside the Ubuntu Mate 18.04 operating system.

### 2.2. Drone Sensors

Angular position information is required to stabilize the UAV. This can be provided indirectly from sensors measuring other values. Popular solutions include linear acceleration sensors (accelerometers) and angular velocity sensors (gyroscopes). To obtain the angular position from the accelerometer, the ground acceleration is used. Depending on the position of the accelerometer, it will be distributed across the axes. This requires establishing a base position where the object is parallel to the ground. The premise of the measurement is to detect roll or pitch by determining the deviation from the axis of gravity, which correlates with acceleration due to gravity. The disadvantage of this solution is the influence of other forces that generate linear acceleration. The noise caused is a high-frequency signal. To determine the angular position from the gyroscope, it is necessary to integrate the data obtained from this sensor. Before starting the integration, the angular position must be given as the initial condition for integration. During the process of computing, the data, accumulating small errors (resulting from integration) create a bias of the gyroscope. As a result, as time passes, the calculated outcome may deviate from the true value.

The following sensors are implemented on board the flight controller:

1. L3GD20;
2. MPU6000;
3. LSM303D;
4. MS5611.

L3GD20 [19] is a low power three-axis gyroscope. It is produced by ST microelectronics for navigation systems. The maximum sensitivity is 8.75 mdps (mili degrees per second)/digit. 

MPU6000 [20] is used as main accelerometer and secondary gyroscope. Depending on the range of measured acceleration, the accuracy is from 2.048 to 16.384 lsb (least significant bit)/g (acceleration expressed in relation to the acceleration due to gravity). The gyroscope in this module is used secondarily. The measurements are used to verify the data from the master module and, in case of discrepancies, represent the reason for rejecting particular samples.

LSM303D [21] is three-dimension accelerometer, used as a secondary 3D magnetometer. A magnetometer is needed for calculation of the yaw angle.

MS5611 [22] is a barometric pressure sensor, the measurements of which are not relevant to the content of this paper.

## 3. Types of Filters

To obtain angular position, intermediate data are used by measuring other physical values. From the accelerometer readings, the deviation from the vertical position is determined by measuring the acceleration of the earth. From the trigonometric functions, the pitch and roll of the measurement system relative to the earth system are calculated. When other linear accelerations are involved, the ratio of the contribution of the ground acceleration to the individual accelerometer axes may be disturbed. This effect is called accelerometer noise. To obtain the angular position from the gyroscope readings, the collected data corresponding to the angular velocity in the respective axes must be integrated. The error that occurs in this case is called gyro drift. It is due to the integration process and imperfections in the system, which, with prolonged operation, begin to accumulate with the integration process. To compensate for these errors, digital filters are used (i.e., the complementary filter and the Kalman filter). The authors of this paper chose to consider these two mentioned filters due to their popularity in flight controllers.

### 3.1. Complementary Filter

**Figure 5:**
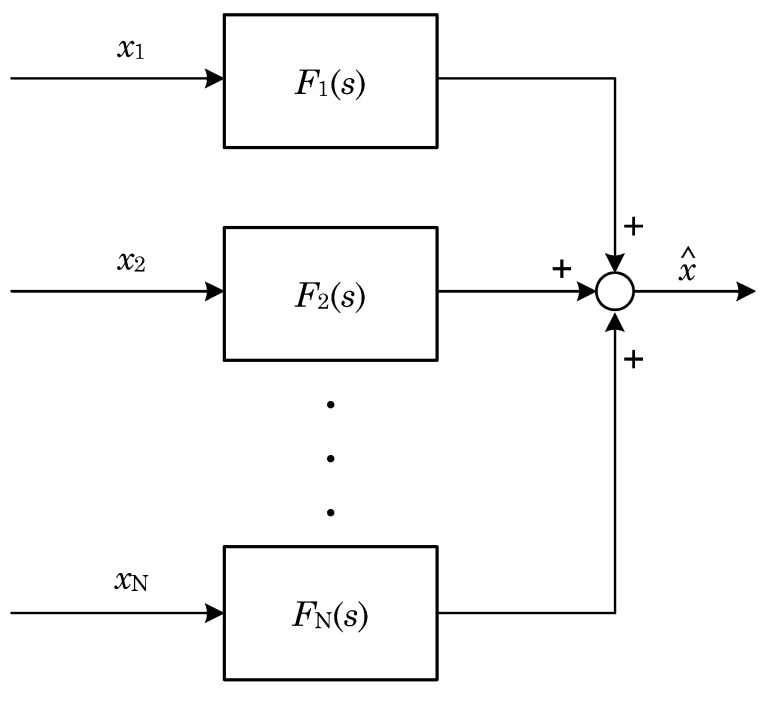
Summation of individual component signals.

The complementary filter is used when data come from different channels and sensors [10]. To obtain the final result, part of the data is added (Figure 5). For every single component, a specified filter (such as LPF (low-pass filter); HPF (high-pass filter)) is used.

The specificity of the filter can be described by the formula:

(3) $$\overset{N}{\underset{i=1}{\sum }}F_{i}\left(s\right)=1$$

**Figure 6:**
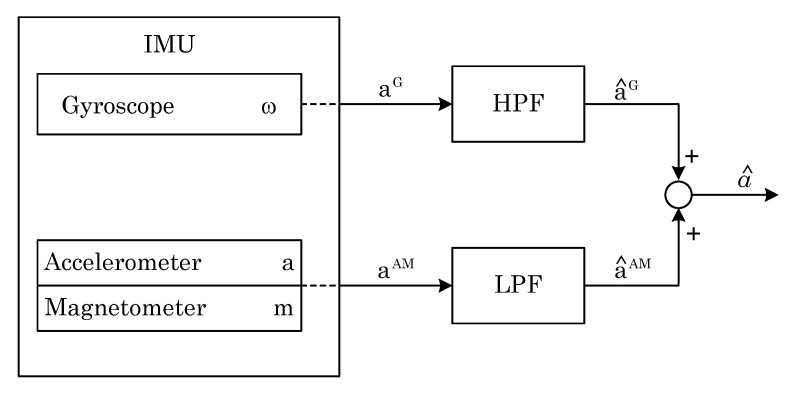
Processing of the IMU sensor data by the complementary filter.

To obtain the angular position of the drone, the data from two independent channels are used (Figure 6). First, data comes from the gyroscope. The obtained value *ω*, which is the angular velocity, is integrated to the angular position. Then, it is passed through a high-pass filter. Secondly, data come from accelerometer and magnetometer. The pitch and roll are calculated from the acceleration values *a* (as described in Section 2.2), while the yaw is determined from the reading of the earth’s magnetic field *m* [11]. The data are then passed through a low-pass filter.

The LPF transfer function can be represented as:

(4) $$H_{LPF}\left(s\right)=\frac{1}{1+T\cdot s}$$

where *T* is the time constant of inertial component and *s* is the complex variable. Analogously, according to the principle of the complementary filter, the HPF transfer function can be written as:

(5) $$H_{HPF}\left(s\right)=1-H_{LPF}\left(s\right)=\frac{T\cdot s}{1+T\cdot s}$$

**Figure 7:**
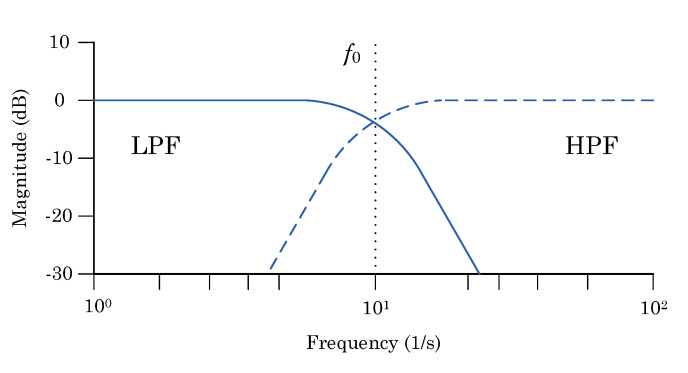
Cut-off frequency for complementary filter.

The parameter for the complementary filter is *f*_0_ which is defined as the cut-off frequency. It determines frequencies for both the low-pass filter and high-pass filter (Figure 7). 

As may be noted in the figure above, by setting a boundary for one basic filter, it is necessary to apply it to the other as well. The optimization of the complementary filter comes down to determining this boundary.

### 3.2. Kalman Filter

The Kalman filter is an algorithm used for multiple measurements that contain inaccuracies, such as statistical noise observed over time. It produces estimates of unknown variable that are more accurate than results based on measurements from a single channel. From the time of its invention until now, the Kalman filter has been refined. There are now many versions of the Kalman filter that are the subject of scientific research [3].

The algorithm operates in a two-steps process. Considering the system as a discrete-time model in state space, the following equations can be written [5]:

(6) x(t+1)=A·x(t)+B·u(t)+v(t)

(7) y(t)=C·x(t)+w(t)

where:

x(t)—state of the system at time *t*,

A—state matrix,

B—input matrix,

v(t)—process noise,

y(t)—system output,

C—output matrix,

w(t)—measurement noise.

The first step is called the time update and it consists in computing the single state prediction (i.e., the a priori estimate and its covariance).

(8) $$\hat{x}\left(t+1\right)=A\cdot \hat{x}\left(t\right)+B\cdot u\left(t\right)$$

(9) $$P\left(t+1\right)=A\cdot P\left(t\right)\cdot A^{T}+V$$

where:

$\hat{x}\left(t\right)$—a priori estimate,

P(t)—covariance matrix for $\hat{x}$,

*V*—covariance matrix for v.

In the next step, the time is increased by one and the algorithm goes on to update the measurement.

(10) $$\varepsilon \left(t\right)=y\left(t\right)-C\cdot \hat{x}\left(t-1\right)$$

(11) $$S\left(t\right)=C\cdot P\left(t-1\right)\cdot C^{T}+W$$

(12) $$K\left(t\right)=P\left(t-1\right)\cdot C^{T}\cdot S^{-1}\left(t\right)$$

(13) $$\hat{x}\left(t\right)=\hat{x}\left(t-1\right)+K\left(t\right)\cdot \varepsilon \left(t\right)$$

(14) $$P\left(t\right)=P\left(t-1\right)-K\left(t\right)\cdot S\left(t\right)\cdot K^{T}\left(t\right)$$

where:

*W*—covariance matrix for *w*,

*ε*(*t*)—the difference between the most recent measurement and the value expected from state estimate,

*S*—covariance matrix for *ε*,

K(t)—Kalman gain.

**Figure 8:**
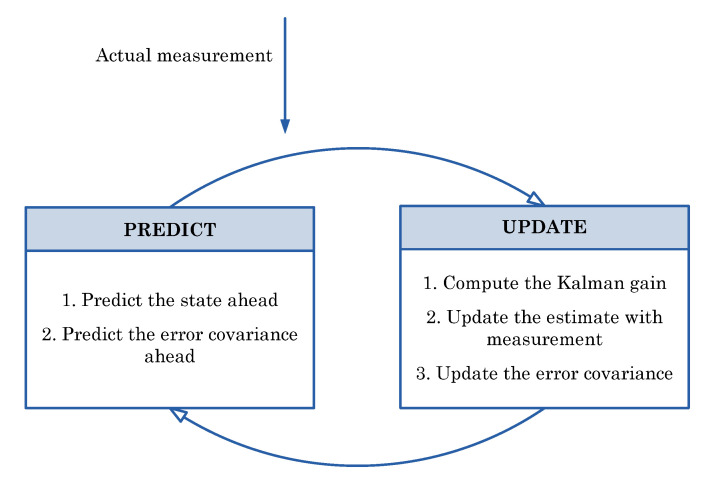
Two steps Kalman filter algorithm.

Kalman gain decides what effect a new measurement has on the a posteriori estimate of the state versus the a priori estimate. The algorithm repeats its steps alternately. This is shown in Figure 8 [7].

Due to the nonlinearity in the measuring system associated with the readout of the speed data, an EKF (extended Kalman filter) is a frequently used form of the Kalman filter. It is presented in detail in [23]. For a nonlinear system, the matrices appearing in Equations (6) and (7) are replaced with a set of nonlinear functions. The equations then have the following form:

(15) x(t+1)=f[x(t),u(t)]+v(t)

(16) y(t)=h[x(t),u(t)]+w(t)

where:

u(t)—input vector,

f,h—sets of nonlinear functions describing state and input dependencies.

In the case under consideration, the input vector is a measurement vector and can be represented as [6]:

(17) $$z_{k}={\left({\;}^{b}a_{t}\;{\;}^{b}w_{t}\right)}^{T}\;$$

where:

${\;}^{b}a_{t}$—the measurements of the linear accelerations, obtained from a triaxial accelerometer,

${\;}^{b}w_{t}$—the three angular velocity measurements obtained from a triaxial rate-gyro.

Functions f,h must be differentiable after all parameters. This makes it possible to linearize the equations. In order to carry out this process, the above-mentioned functions should be developed into Taylor series. It is assumed that the obtained matrices *A*, *B*, *C*, *D* approximate the behavior of the studied nonlinear system in the neighborhood of the given operating point (*x*’, *u*’). To obtain the desired matrices, it is necessary to determine the partial derivatives of the functions from the nonlinear model:

(18) $$A={\frac{\partial f}{\partial x}|}_{x=x’,u=u’}$$

(19) $$B={\frac{\partial f}{\partial u}|}_{x=x’,u=u’}$$

(20) $$C={\frac{\partial h}{\partial x}|}_{x=x’,u=u’}$$

(21) $$D={\frac{\partial h}{\partial u}|}_{x=x’,u=u’}$$

The EKF equations differ slightly from those of the linear Kalman filter and can be expressed as follows:

(22) $$\hat{x}\left(t+1|t\right)=f\left[\hat{x}\left(t|t\right),u\left(t\right)\right]$$

(23) $$P\left(t+1|t\right)=A\left(t|t\right)\cdot P\left(t|t\right)\cdot A^{T}\left(t|t\right)+V$$

Equations (10)–(14) corresponding to state updates can be reformulated as follows:

(24) $$\varepsilon \left(t\right)=y\left(t\right)-h\left[\hat{x}\left(t|t-1\right),u\left(t\right)\right]$$

(25) $$S\left(t\right)=C^{T}\left(t|t-1\right)\cdot P\left(t|t-1\right)\cdot C\left(t|t-1\right)+W$$

(26) $$K\left(t\right)=P\left(t|t-1\right)\cdot C^{T}\left(t|t-1\right)\cdot S^{-1}\left(t\right)$$

(27) $$\hat{x}\left(t|t\right)=\hat{x}\left(t|t-1\right)+K\left(t\right)\cdot \varepsilon \left(t\right)$$

(28) $$P\left(t|t\right)=P\left(t|t-1\right)-K\left(t\right)\cdot S\left(t\right)\cdot K^{T}\left(t\right)$$

These equations are well known. Detailed considerations for the EKF can be found in the publications [4,23].

## 4. Data Processing

The MATLAB 2021a environment was used for data processing. Archived data were collected to 12 precision decimal places. Ready-made filters available from version 2019b were used to process the data. The arbitrary values are the data obtained from the measuring station. 

The test procedure carried out in this paper to obtain the optimal filter and to improve the embedded filter included the following operations:

- Collection of position data along with arbitrary data;
- Obtaining position data from the readout of the sensors themselves;
- Data processing with complementary filter and EKF;
- Filter tuning;
- Comparison of the prepared results with the filter built in PX4 and arbitrary data;
- Update of the built-in filter (EKF coefficients);
- Repeating the experiment and collecting data;
- Comparison of the performance of the built-in filter after updating the coefficients.

### 4.1. Raw Data

**Figure 9:**
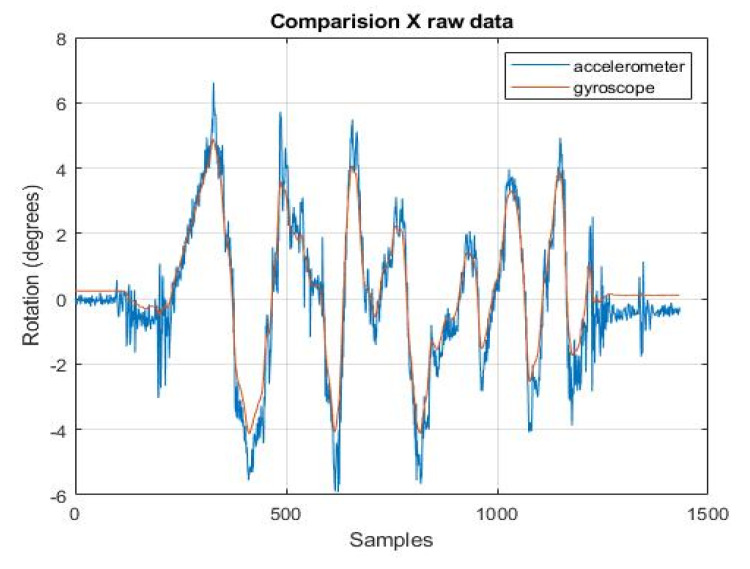
*x*-axis rotation readings from accelerometer and gyroscope.

**Figure 10:**
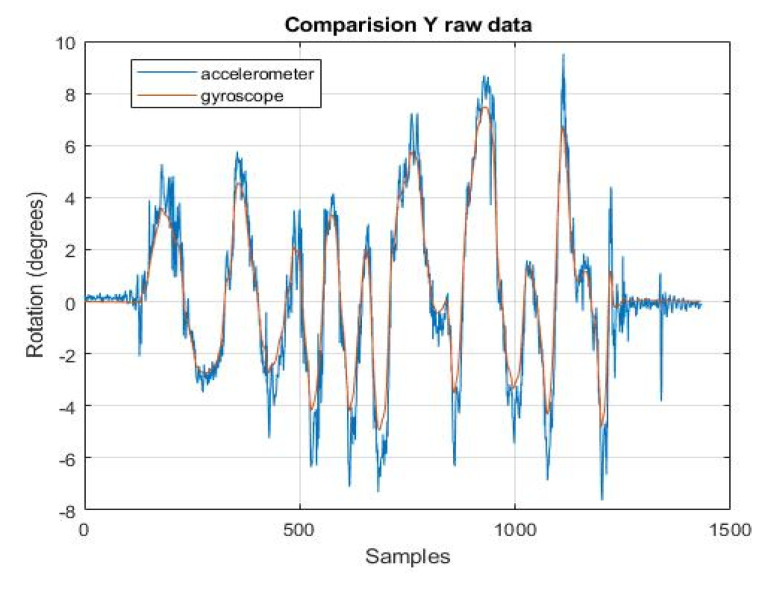
*y*-axis rotation readings from accelerometer and gyroscope.

Figure 9 and Figure 10 show the angular position calculated from the data from the individual sensors. It can be noticed that the position obtained from the accelerometer looks much noisier in comparison to the position from the gyroscope. There are a lot of peaks in the figure, and the estimation run is very dynamically changed. It was assumed that the initial position of the gyroscope, necessary for the integration process, should be taken from the accelerometer.

Both figures above are taken from the raw data. They are provided to show how the angle estimation task is handled by the various sensors. In the next steps, the obtained estimates will be compared with the results obtained from the filters.

### 4.2. Complementary Filter

**Figure 11:**
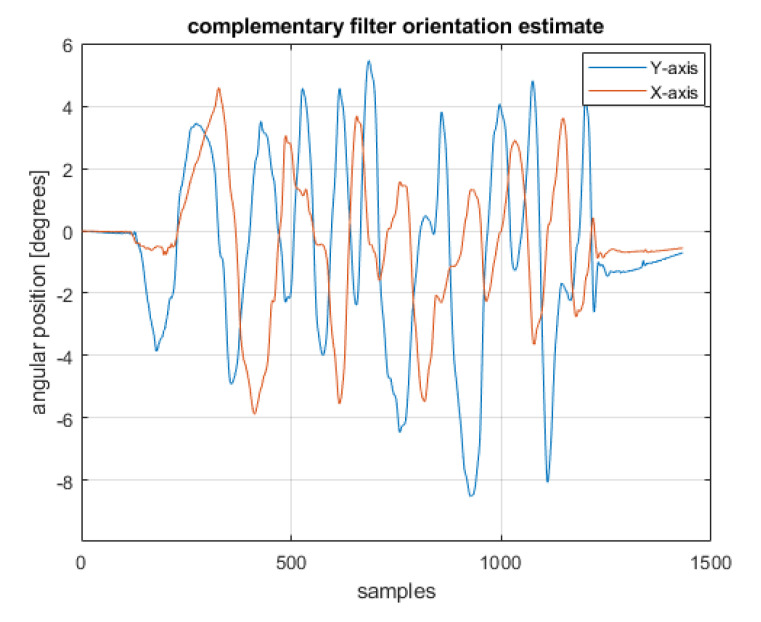
Position estimation using the complementary filter.

The complementary filter was designed using the ready-made ‘complementaryFilter’ object [24]. this corresponds to the theoretical considerations described in Section 3.1. The data obtained from the filter were converted to Euler angles and are presented in Figure 11.

### 4.3. Extended Kalman Filter

**Figure 12:**
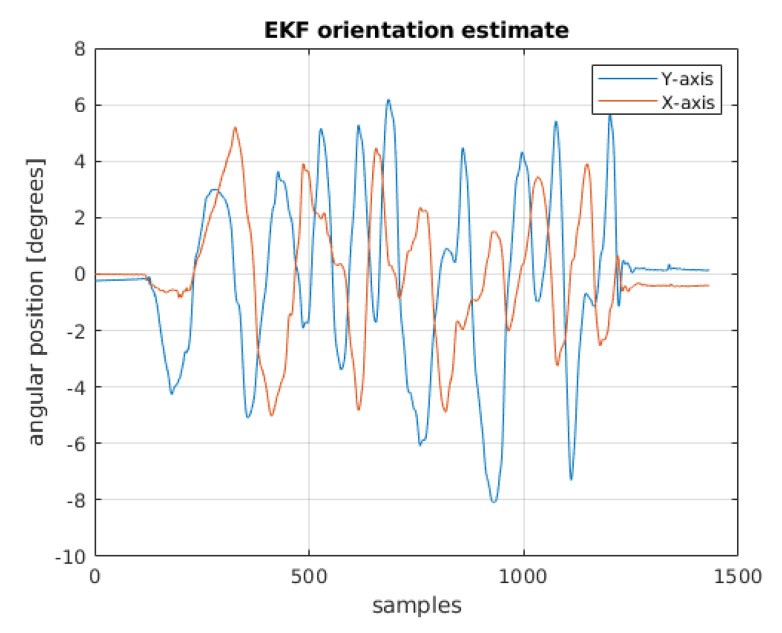
Position estimation using the EKF.

The EKF was designed using the ‘insfilterNonholonomic’ object [25]. This object is a specialized version of the EKF, dedicated to work with data coming from IMU sensors. It has a ‘tune’ method allowing one to optimize the filter parameters referring to arbitrary data. The data obtained from this filter are shown in Figure 12.

### 4.4. Comparison between Filters

**Figure 13:**
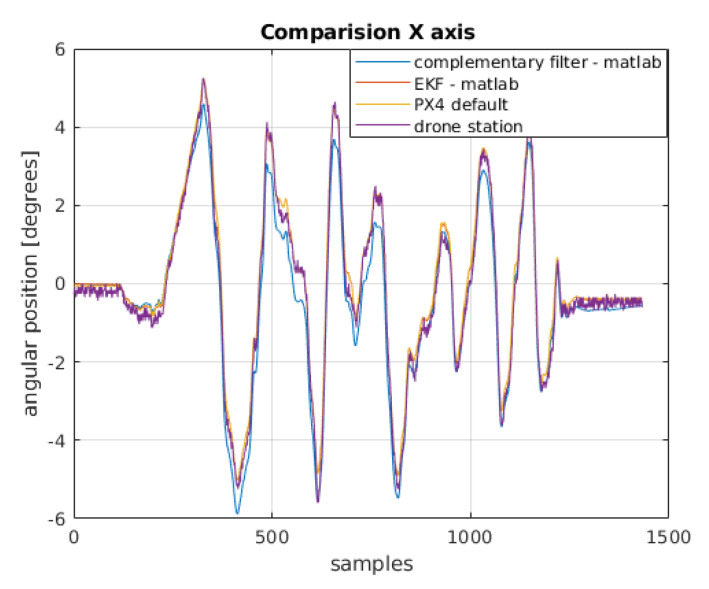
Comparison of the angular position obtained from the filters with the physical position for the *x*-axis.

**Figure 14:**
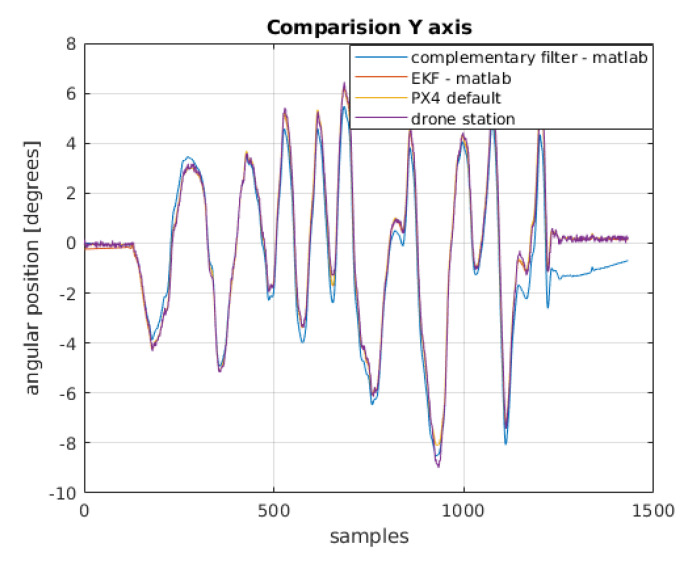
Comparison of the angular position obtained from the filters with the physical position for the *y*-axis.

To compare the filters, apart from those designed in MATLAB environment, a default filter implemented in PX4 software is presented. It is an extended Kalman filter. In Figure 13 and Figure 14, a summary of these filters together with the arbitrary position obtained from the test-bench may be seen.

## 5. Results

The criterion adopted to evaluate the filters is the integral absolute error (IAE). Estimations from raw data from both sensors, a complementary filter, an EKF implemented in MATLAB, and an EKF from PX4 software were evaluated. The results are presented in Table 1.

**Figure 15:**
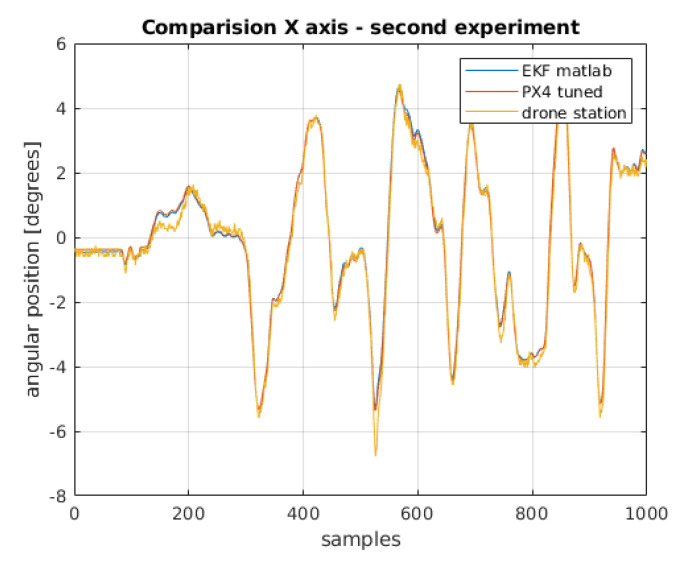
Comparison of angular position for *x*-axis.

**Figure 16:**
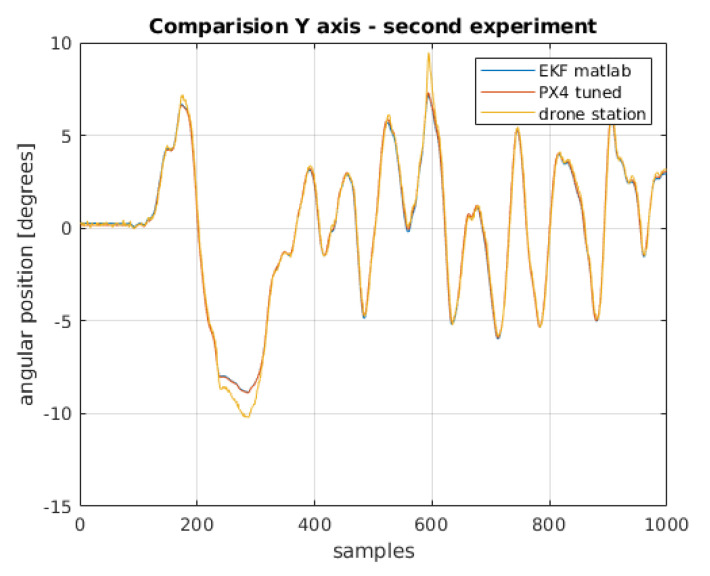
Comparison of angular position for *y*-axis.

Definitely the worst in the comparison are the results estimated only on the basis of the reading from the accelerometer. This is due to the accelerometer noise. A significant improvement can be seen in the results obtained from integrating the gyroscope readings. It should be recalled that the initial condition for integration (i.e., the initial position of the drone) was assumed on the basis of the first reading from the accelerometer. Without this, obtaining the angular position would have been impossible, or it would have been necessary to assume that the drone always takes off from a specific position, which in reality is not very realistic. The best physical position was calculated by extended Kalman filters. The extended Kalman filter implemented in the PX4 firmware contains process and measurement noise covariance matrices that do not take into account the drone’s physical parameters. These parameters can be changed in the configuration structure when uploading the software to the flight controller. This requires a change in the source code and a build for a specific controller model. A simpler method is to find and change the desired parameters in the QGroundControl software dedicated to the configuration of flight controllers running under PX4 control. During the research work, QGroundControl software version 4.1.1 was used. To compare the operation of filtering algorithms in the extended Kalman filter, parameters selected by the tune function from MATLAB [26] were uploaded to the flight controller. The bench test was carried out again. The results shown in Figure 15 and Figure 16.

After transferring the filter parameters from the MATLAB script to PX4 software, running the experiment on the test bench, re-archiving the data, processing the raw readings, and filtering with the previously designed EKF, there is no difference in the comparison of the estimation between the filter built in PX4 software and the filter designed in the MATLAB environment. 

The integral absolute error values are close to being the same Table 2. The difference in values is less than 1% and can be considered negligible.

## 6. Discussion

The paper presents a comparison of available methods of filtering signals from the most popular sensors used in unmanned aerial vehicles. Its purpose was to develop and investigate an algorithm for improving angular position filtration in a real drone. The research was conducted on a drone that can be self-assembled from commonly available and affordable components. As can be seen from Table 1, using only one sensor is not sufficient to obtain reliable data. It is necessary to use filtering. The authors of this study showed that the extended Kalman filter is clearly preferable to the complementary filter. It reproduces the course of changes in the angular position of the real drone much better. Only during the biggest angular changes in peaks was the complementary filter closer to the real value. As can be seen from the data presented in Table 1, the EKF after tuning in MATLAB has the smallest IAE error. The development of embedded systems has made the extended Kalman filter, despite its greater computational complexity, a solution that does not overload the processing power of flight controllers.

Other researchers may use this work to practically improve the filter performance in their drones by reflecting drone station and our procedure presented at the beginning of Section 4. The authors recommend that anyone wishing to take advantage of the presented results perform the individual steps on their own object. Due to the physical specifications of the real quadrotor object, including sensors, it is not suitable to compare the results between different objects or between object and simulation. The MATLAB program was used to process the data collected from the real object. Then, based on multiple iterations of filtering processes on the same data, the best optimized filter was selected from those available in the flight controllers. A further research direction projected by the authors is to study the optimization of individual flying units and the effect of unit improvement on swarm behavior during a cooperative task of multiple UAVs.

## 7. Conclusions

This article shows how the filter embedded in the flight controller software can be improved. Popular flight controller software such as Ardupilot and PX4, have implemented filters whose parameters can be changed by the user. Based on arbitrary angular position data, a “tune” function was used to optimize the filter coefficients. Angular position estimation of the real drone has been improved, which was our goal.

## Figures and Tables

**Table 1:** Quality indicator.

	IAE for *x* axis	IAE for *y* axis
Gyroscope	704.6	628.9
Accelerometer	3897.8	4487.6
MATLAB complementary filter	489.4	472.5
MATLAB EKF	291.3	232.6
PX4 EKF	304.8	276.4

**Table 2:** Quality indicator.

	IAE for *x* axis	IAE for *y* axis
MATLAB EKF	238.3	311.0
PX4 EKF tuned	239.5	313.3

## Data Availability

The dataset used and analyzed in this study are available from the corresponding author upon reasonable request.
